# “It was an important part of my treatment”: a qualitative study of Norwegian breast Cancer patients’ experiences with mainstreamed genetic testing

**DOI:** 10.1186/s13053-022-00212-6

**Published:** 2022-02-05

**Authors:** Nina Strømsvik, Pernilla Olsson, Berit Gravdehaug, Hilde Lurås, Ellen Schlichting, Kjersti Jørgensen, Teresia Wangensteen, Tone Vamre, Cecilie Heramb, Lovise Mæhle, Eli Marie Grindedal

**Affiliations:** 1 Department of Health and Caring Sciences, Western Norway University of Applied Sciences, Bergen, Norway; 2 Northern Norway Familial Cancer Center, Department of Medical Genetics, University Hospital of North Norway, Tromsø, Norway; 3 Department of Surgery, Section of Breast and Endocrine Surgery, Innlandet Hospital, Hamar, Norway; 4 Department of Breast and Endocrine Surgery, Akershus University Hospital, Lørenskog, Norway; 5 Health Services Research Unit, Akershus University Hospital, Lørenskog, Norway; 6 Institute of Clinical Medicine, University of Oslo, Oslo, Norway; 7 Section for Breast and Endocrine Surgery, Department of Cancer, Oslo University Hospital, Oslo, Norway; 8 Department of Medical Genetics, Oslo University Hospital, Oslo, Norway

**Keywords:** Breast cancer, *BRCA1*, *BRCA2*, Mainstreamed genetic testing, Qualitative study, Genetic testing for breast cancer

## Abstract

**Background:**

In South-Eastern Norway, genetic testing for *BRCA1* and *BRCA2* is offered to breast cancer patients by their treating surgeon or oncologist. Genetic counselling from a geneticist or a genetic counsellor is offered only to those who test positive for a pathogenic variant or have a family history of cancer. This practice is termed “mainstreamed genetic testing”. The aim of this study was to learn about patients’ experience of this healthcare service.

**Methods:**

Qualitative in-depth interviews were conducted with 22 breast cancer patients who had been diagnosed during the first half of 2016 or 2017 at one regional and one university hospital and who had been offered testing by their treating physician. A six-phase thematic approach was used to analyse the data.

**Results:**

The participants had varied experiences of how and when testing was offered. Three main themes emerged from the analysis: 1. informational and communicational needs and challenges during a chaotic time, 2. the value of genetic testing and 3. the importance of standardised routines for mainstreamed genetic testing.

**Conclusions:**

Despite the shock of their diagnosis and the varying experiences they had in respect of how and when testing was offered, all of the participants emphasised that genetic testing had been an important part of their diagnosis and treatment. Our results indicate that there is a need for continuous collaboration between geneticists, surgeons, oncologists and laboratory specialists in order to establish simple and robust routines so as to ensure that all eligible breast cancer patients are offered testing at a point when the test result can have an impact on treatment.

## Background

Women with inherited pathogenic variants in the *BRCA1* and *BRCA2* genes have a high risk of developing both breast and ovarian cancer. Their cumulative risk of breast cancer (BC) may be as high as 70% for carriers of both genes, whereas the risk of ovarian cancer (OC) is estimated to be 44% for *BRCA1* carriers and 17% for *BRCA2* carriers [1].

Identifying a pathogenic *BRCA* variant in a woman who has been diagnosed with BC provides important information for making both oncological and surgical treatment decisions regarding her current cancer [2–4]. In addition, breast and ovarian cancer may be prevented from developing through a risk-reducing mastectomy and salpingo-oophorectomy in the patient and in relatives who may also carry the variant [5].

For many years, due to high costs, genetic testing was offered only to BC patients with a high risk of being a carrier; i.e. they were young (< 50 years) at the time of diagnosis or had a significant family history of breast and/or ovarian cancer. Testing was ordered by medical geneticists or genetic counsellors after genetic counselling and usually after the completion of treatment. Recent years have seen great advances in the technology of gene sequencing, leading to increased capacity, shorter turn-around time in the lab, and reduced costs. These changes have made it feasible to offer testing to a greater number of patients. In addition, due to the growing role of the genetic test result in treatment decisions [6–8], carrying out the testing at the time of diagnosis or during primary treatment has become more clinically relevant and important.

Some early studies have argued that offering testing at the time of diagnosis or during treatment may be too burdensome for the patient: patients may already be emotionally overwhelmed with their situation, making it difficult for them to make an informed decision about testing and to understand the implications of a positive test result for themselves and their families [9, 10]. Since the publication of these results, awareness of the heritability of BC and genetic testing has grown in the general population and among BC patients; therefore the results of these early studies may no longer be valid for BC patients who are being diagnosed today. More recent studies have not reported the same results. These newer studies have demonstrated that offering genetic testing shortly after diagnosis does not cause adverse psychological effects in BC patients [11, 12]. Correspondingly, qualitative studies have shown that patients who were offered genetic counselling and testing before primary treatment felt that it was highly relevant when making decisions about surgery, which suggests that testing should be offered shortly after diagnosis [13–15]. In most of the studies to date reporting on this subject, patients have been offered both genetic testing and counselling and/or testing has been done as part of a research protocol. This may affect results variously: participants may receive more information or attention in research studies than in clinical care, which in turn may affect their responses. In addition, research studies that include genetic testing as a recruitment tool may attract patients who are initially more positive about testing [16].

In South-Eastern Norway, genetic testing of BC patients was incorporated into regular clinical care in hospitals beginning in 2014, a model often referred to as “mainstreamed genetic testing”. The treating surgeon or an oncologist can offer genetic testing to BC patients who fulfil the criteria issued by the Norwegian Breast Cancer Group (NBCG) [17]. The physician provides the patient with information about genetic testing based on standardised written information. However, the guidelines do not state when during diagnostics and treatment the testing should be offered. Since 2014, testing has included BRCA1 and BRCA2, and in spring 2020 PALB2 was also added. Patients are referred for genetic counselling only if a pathogenic variant or a variant of uncertain clinical significance (VUS) is detected. Patients who have a normal test result but have been diagnosed with BC aged 40 years or younger or who have a family history of cancer are also referred for genetic counselling and offered testing for other cancer predisposition genes at the regional genetics department at Oslo University Hospital (OUH). The panel currently in use in South-Eastern Norway includes 29 highly and moderately penetrant BC genes as well as genes that create a predisposition to other types of cancer such as colorectal and prostate cancer. The aim of mainstreaming genetic testing into regular clinical care has been both to make testing available to more BC patients and to be able to use the test result to guide treatment. We have recently reported that in two hospitals in South-Eastern Norway 75% of the patients eligible for testing according to the Norwegian guidelines were offered testing [16]. This is a higher rate of testing than in other countries [18–20]. We also found that 96% of those who were offered a test wanted to be tested [16].

While our previous study demonstrated that genetic testing is offered to most of the BC patients who are eligible for it, we have limited knowledge of their experience of being tested as part of regular clinical care shortly after diagnosis or during treatment and without receiving genetic counselling. We have therefore carried out a qualitative study with individual in-depth interviews of BC patients who have been offered genetic testing by their treating oncologist or surgeon at two hospitals in South-Eastern Norway. Our aim was to acquire a broad range of information about their experience with this healthcare service that can be used to improve its quality in terms of access to testing and how and when the offer is made.

## Methods

### Participants and procedures

We have recently conducted a study of rates of genetic testing among BC patients diagnosed during the first half of 2016 or 2017 at one regional and one university hospital in South-Eastern Norway [16]. Of those who consented to inclusion in this study, 30 patients of various ages who fulfilled the NBCG criteria for genetic testing and underwent testing during BC treatment were sent an invitation to take part in the qualitative study. The patients were selected irrespective of their genetic test results. Women who wished to participate returned the consent form and were contacted by telephone to arrange an interview. They could choose to be interviewed at home, in one of the hospitals or at another location which they preferred. No reminders were sent to those who did not respond to the invitation letter. All but one of the patients had finished their treatment by the time the interviews took place. The interviews were conducted in Norwegian and all of the participants spoke Norwegian fluently.

All of the women who had agreed to participate in the study were interviewed, although saturation was achieved prior to all participants being interviewed.

### Interviews

In-depth individual interviews were performed during the fall of 2019 by the first and last authors, both of whom are genetic counsellors with long-standing experience in counselling for hereditary cancer. The interviews were based on an interview guide consisting of open themes. A former BC patient who agreed to serve as a user representative was actively involved and commented on the guide before the interviews took place.

Initially, the women were encouraged to relate their experience of their cancer diagnosis, including how and when they were offered genetic testing. We also wished to further explore the patients’ experience of the genetic testing process. In most of the interviews, this generated a story about the emotional distress of being diagnosed with BC, the participant’s cancer treatment and their genetic testing. If not already part of their story, they were asked about the timing of the genetic testing, how information about it was presented to them and their experience of this process. The participants were also asked about their attitudes towards the genetic testing of BC patients in general; they were encouraged to address issues of importance to them. The mean duration of the interviews was 41 min and 45 s (range: 18:42–1:38:49).

### Data analysis

All of the interviews were audio recorded and transcribed verbatim. A six-phase thematic analysis as set out by Braun and Clarke [21] was then used to analyse the interviews:

1. Familiarisation with the data by reading and re-reading the interviews.
2. Production of initial codes from the data.
3. Sorting the different codes according to potential themes.
4. Reviewing the themes.
5. Defining and naming the themes.
6. Writing the paper.

The first and last authors read the interviews repeatedly and took notes. The first and last authors also discussed and defined the formulation of themes, which they identified during the main coding process. Continual transition between the various phases was allowed for throughout the analysis process, which also included writing the paper. The user representative was consulted during this process and the themes were discussed with the co-authors.

The computer program NVIVO was used to organise and code the processing of data.

## Results

### Sample

Of the 30 women invited, 23 (76.6%) initially consented to be included. One patient later cancelled her interview. Therefore, individual in-depth interviews were conducted with 22 patients (73.3%), 11 from the university hospital and 11 from the regional hospital. One participant had tested positive for a *BRCA2* pathogenic variant. The remaining patients had received normal test results. See Table 1 for more detailed information about the sample.

**Table 1:** Participants

16 women from the university hospital and 14 women from the regional hospital were invited to participate. 11 women from the university hospital and 11 women from the regional hospital participated.	22/30 (73%)
**When genetic testing was offered to the patient and by whom**	**Participants (*n*** **= 22)**
By surgeon before surgery	8 (36.4%)
By surgeon after surgery	5 (22.7%)
By oncologist during neo-adjuvant therapy	4 (18.2%)
By oncologist during adjuvant therapy	3 (13.6%)
During radiation treatment	1 (4.5%)
Could not remember	1 (4.5%)
**Asked for the test themselves**	6 (27.3%)
**Time from diagnosis to interview**	Range: 2–4 years (mean 3)
**Age at diagnosis**	Range: 35–64 years (mean 48.7)
**Age at interview**	Range: 38–66 years (mean 51.6)
**Results from genetic test**	One positive for a BRCA2 pathogenic variant 21 normal test results

### Ethics approval and consent to participate

The study was defined as a quality-of-care study and approved by the data protection officers at the two hospitals included in the study and at OUH.

All participants consented to inclusion in the study and were able to withdraw at any time. Audio files were stored on the secure server of OUH for deletion upon completion of the study. All participants are pseudonymised in the present material. Quotes have been modified to improve flow without changing the content.

### Results of the thematic analysis

The main objective of this study has been to investigate the BC patients’ experience of mainstreamed genetic testing. This experience was closely linked to and affected by their experience of being diagnosed with and treated for BC itself; hence this too is to some extent included in this thematic analysis. Three core themes and several subthemes have been identified. The themes include: (1) informational and communicational needs and challenges during a chaotic time, (2) the value of diagnostic genetic testing (3) and the importance of standardised routines for mainstreamed genetic testing.

### Theme 1: informational and communicational needs and challenges during a chaotic time

Information and communication were recurrent themes in many of the women’s experience of BC diagnosis, treatment and genetic testing. The participants described in detail the shock and acute psychological stress of being diagnosed with BC and how it simultaneously created a need for information and made it difficult to absorb and remember information. Their experience with information and communication regarding genetic testing were closely linked to how information and communication regarding breast cancer was perceived. A feeling of trust in their healthcare providers facilitated communication.

#### Subtheme: the shock of being diagnosed with breast cancer created both a need for information and an obstacle to absorbing information

The participants underwent different forms of BC treatment, ranging from breast-conserving surgery to combined surgery, chemotherapy and radiation treatment. Despite these differences, many of them said that being diagnosed with BC was a shocking and overwhelming experience. “Susan*”* described the day she received her diagnosis as follows:

*[A] nd they took a biopsy, and then I came to the surgeon, and I don’t remember anything of that really, and what happened there. And then I left the hospital like some sort of a mummy, I think, with the certainty that I had cancer.*

Many expressed being overwhelmed by fear and insecurity. They went into their own bubble filled with worry about their own life and future and for their daughters or sisters. Many described themselves as having a “*cotton-head*”. They talked about receiving a lot of information about their diagnosis and treatment and how it was difficult, almost impossible, for them to take in and remember information. As *“*June*”* puts it:

*When you are in the middle of a situation like that, maybe you have just had surgery, you have many thoughts in your head, and you don’t remember what’s been said to you. I hardly remember anything from the day I was diagnosed. I just remember that I felt really sad.*

Some of the women reported avoiding information, but many said that despite their difficulties remembering, they still felt a great need for information in order to navigate this new situation. However, this information had to be presented by people they trusted. Finding information on the internet could often lead to more questions and fear. Information was seen as a way of gaining control over the situation and reducing their fear and insecurity. “Anne”, who was 41 at the time of her diagnosis, described her experience as follows:

*I was very clear with them that I wanted to know all I could know, and that I didn’t know anything. They were very clear about telling me “Don’t Google,” but then I said, “Then you have to answer all my questions, because otherwise I will Google,” and you know that when you Google you always end up finding scary things or negative things or … .*

#### Subtheme: informational needs and challenges regarding genetic testing

Some of the participants had been thoroughly informed by their doctor about the consequences of a positive test result in terms of the risk of a new case of breast and/or ovarian cancer. They valued this conversation, the way the surgeon or oncologist communicated with them and the fact that the doctor had taken the time to discuss it with them. However, most of the participants said either that they could not remember being informed specifically about these issues or that they had not been informed. They knew that the aim of the test was to investigate whether their cancer was hereditary but they had not been informed of any other details. Most of these participants said that they were happy and content with the way the test had been offered, with some emphasising that the most important thing was being offered the test. As *“*Joan*”* states:

*They just asked whether I was interested in knowing whether it was a hereditary form of cancer that I had. And there was no doubt in my mind. Of course, I wanted to know …*. *So, it was fine. It was no problem.... I didn’t feel.... I just thought it was a good thing … that they actually brought it up.*

A few of the participants felt they had not been given enough information about the test. These women also expressed general frustration with the lack of information about their diagnosis and treatment.

*“*Sylvia*”* described feeling like she was on an assembly line where there was no time to inform her the way that she felt she needed to be informed:

*To me it [the information about genetic testing] was just a small sentence in the information about chemotherapy and the plan for the rest of my treatment. It was. Because the only thing I was informed of when I had surgery was that because you have had a lumpectomy you will have five weeks of radiation therapy”.*

At the same time, it was hard for her to identify the kind of information she missed out on because, as she puts it:

*The ignorant doesn’t know what to ask about.*

When asked what information about genetic testing they thought should be provided and how it should be provided, two of the participants said that they did not want information about potential consequences before it became a reality. However, a majority of the participants thought that it would be useful to be informed about what would happen if they tested positive for a *BRCA* mutation. At the same time, they were all aware of their own challenges and limitations when it came to remembering information while they were in such a chaotic state and that it was difficult to provide patients with information at this stage. *“*Mary*”* puts it like this:

*It is important to be aware that the patient is not thinking clearly.*

Because of the difficulties they had remembering what had been said to them, they emphasised how important it was to receive written information. Written information was perceived as a safety net and something they could always go back to. Those who had someone accompanying them to appointments at the hospital emphasised how helpful this had been. The companion was able to help them remember and interpret what had been said.

#### Subtheme: trust and confidence in the healthcare provider facilitates communication

Many of the participants spoke very warmly of the doctors and nurses they met during their treatment. They talked about being seen and met as a person and how important that had been to them. Many of them also described how much they had trusted their surgeon or oncologist’s competence and how this had made them feel safe and reassured. For *“*Elizabeth*”*, the offer of genetic testing itself demonstrated that she could trust in the competence of her doctors:

*I thought it [being offered testing] was a very good thing, and a reassuring thing. The more you feel that doctors know, the safer you feel really.*

For some, this underlying trust also made it easier to communicate and take in information about genetic testing. One participant said she felt completely relaxed after receiving information from her surgeon about genetic testing and the consequences of a positive test result. *“*Sheila*”* felt her surgeon had found the key to communicating about genetic testing:

*My surgeon had the right combination of knowledge, empathy and seriousness, as well as optimism, a smile and warmth. There must be some warmth in there. You cannot be an ice-cold person ... and show that you think this [genetic testing] is difficult. That is a bad solution.*

Some also said that when they trusted their doctors, they did not need detailed information about all aspects of treatment or genetic testing.

### Theme 2: the value of diagnostic genetic testing

All of the participants indicated that genetic testing had been important to them. They saw the test as an important part of their cancer treatment and an opportunity to obtain information that could help protect their children and other close relatives. Some expressed mixed feelings over a normal test result.

#### Subtheme: genetic testing as an important part of cancer treatment

Following the shock and fear and all of the questions associated with a BC diagnosis, the genetic test was regarded as a way of getting more information and clarity within a chaotic situation. Some of the women were aware of the potential consequences a positive test result might have for surgical decision-making, but most of the women did not. The test was nevertheless seen as an opportunity for the doctors to get important information about their tumour that could be used to guide treatment. “Anne” describes it like this:

*To me personally, it was important. I want as much information as possible and as much clarity as possible about …. what this disease means for me.*

Only one of the participants, “Ruth*”*, expressed some mixed feelings about the genetic test:

*I said yes right away. But afterwards I thought, “Oh, I can’t really take any more. Because I am so afraid of the answer that I will get. But I must do this.” So, I thought a bit more about it after I had said yes, but then I thought, “I’ll just do it. It is important that I do it.”*

#### Subtheme: feelings of responsibility for children and other relatives

Most of the participants indicated that their main motivation for undergoing genetic testing was their concern for their close relatives, especially their daughters. They were worried about their relatives’ risk of BC following their own diagnosis: the thought of these relatives, and especially their daughters, being at increased risk of cancer was frightening. For many of the women, these thoughts came shortly after diagnosis. Many of the participants had reflected a great deal upon the different types of impact that a positive test result could have on their daughters’ lives. They said that they saw the genetic test as an opportunity to obtain important information that could protect them, and that the positive effect of being able to prevent cancer clearly outweighed the burden of living with knowledge of the risk. “Melissa” had thought a lot about this:

*If it turns out that it is hereditary, and it turns out that your daughter also has it, then there are some choices she has to make in her life, right? That she has to consider, that are quite serious. But they can do things that will prevent cancer from happening, and they don’t have to go through this treatment, which in a way also can affect the rest of your life. I have talked to others who say, “Why? … Life is a lottery, and if you were to have this hanging over your head it would be a burden if she got to know that she …*.” *So, have I... That has swirled around too, but I think that I would have given her an advantage anyway.*

*“*Hannah*”*, who was identified as having a pathogenic mutation in *BRCA2,* put her thoughts about her children like this:

*And it was ... but I have to say that when I was told that it was a genetic defect, I think it was tough ... and now I might get a bit teary-eyed. But it’s mostly with the children in mind …. And I was afraid they would develop cancer. But then ... [coughs and sniffs], but then they were really ... I really thought we were quite lucky.... Because if they have ... the genetic defect, then they get a much better follow-up than if they had not been tested, right? So, it might prevent their maybe having to go through chemotherapy and things like that … .*

#### Subtheme: mixed feelings over a normal test result

A few of the participants had worried about their results and some thought the waiting time for the results was too long. However, most of the participants did not think it was particularly difficult waiting for the test results because they were mainly occupied with their cancer treatment. As “Mary” puts it:

*There are so many things you think about. How will it be for your family? Will you survive? What will chemotherapy be like? So, the genetic test … I didn’t think about it at all before I had the test results and they were negative. Then I was relieved, of course. But I think it is worse not to get it [the genetic test]. Because then you can wonder for a long time, “What if I had gotten the test? Maybe I would have received another form of treatment? Maybe things would have been different?”*

All but one of the participants had normal test results. They were relieved that they had tested negative, for themselves, of course, but especially for their daughters. Even though they were happy that they did not have a *BRCA1/2* mutation, a couple of the women said that they sometimes wished they had been identified as mutation carriers. If they had tested positive, then they would have been entitled to a risk-reducing mastectomy and would not have had to worry about developing BC a second time. “Melissa” said that it would have helped her to explain why she had developed BC at such a young age.

*For a period I almost wished that it was ... that I had the mutation.... Because then ... then it wasn’t my fault, or I couldn’t have caused it, if you know what I mean. Then it would have been the mutation that caused it.... There were many such thoughts of why in the beginning.*

### Theme 3: the importance of standardised routines for mainstreamed genetic testing

When asked, all of the participants said that every BC patient should be offered genetic testing by their treating physician. Two of the participants said that it should be mandatory, just something that the surgeon or oncologist ordered, but the other participants said that it should be offered with the option to accept or decline. They emphasised the importance of standardised routines to ensure that patients were given the opportunity to be tested, but they had different views about when it should be offered. Some said “the sooner, the better”, whereas others said that it could be too much to take in at the time of their diagnosis. One participant said that it should be offered before chemotherapy because of the negative side effect this treatment has on memory and concentration. However, all of the participants said that if the results would have a bearing on treatment decisions, the offer of a genetic test needed to be given at diagnosis or shortly thereafter. If the test result might have an impact on their treatment, then undergoing the test in good time was the most important issue to be addressed. None of the participants thought that testing should be postponed until after treatment had been completed.

There was great variation in the participants’ stories of how and when they were offered testing, how much information they received before the test and how they received the test results. Most of them had been offered the test but six had asked for it themselves. Two were not sure whether they would have been offered the test if they had not asked for it. “Alyson*”* did not think about it at the time but later she reflected on the fact that she had had to ask for the test:

*In retrospect, I’ve been thinking that when it concerns everyone, and not just me who was so inform, I think it should be an offer that came to me, or at least that you got information that there was a possibility of having a genetic test.*

She recognised that BC patients might not all think the same way about whether or not they wanted to be tested, but she emphasised the importance of having a reliable system for this health service in terms of both the test being offered to BC patients and the information they received.

*“*Melissa*”* was diagnosed with BC at 38 years of age. She had raised the question of genetic testing during radiation treatment and had to ask for the test result six months later. She was very aware of the potential consequences of having an undetected mutation and how important it was to have reliable routines for genetic testing:

*I think that the danger of not having a system for genetic testing is that I might have missed such an offer if I hadn’t been aware of it myself and asked for it. Then I would have learned about it afterwards that it was an offer that I was entitled to, that I didn’t get. To me that would not have been OK. I mean, you want to have all you can get in terms of follow-up and treatment.*

Her experience also led to feelings of uncertainty. It affected her trust in the test results: she feared that the doctor might have misinterpreted the result or maybe did not have enough genetic knowledge. She also said that it negatively affected her trust in the healthcare system more generally:

*It does something to the trust. There is something about the trust in the one who has promised you something, and then it doesn’t happen. It is unfortunately something that affects your trust in other doctors in other situations.*

## Discussion

The BC patients interviewed in this qualitative study of mainstreamed genetic testing revealed varied experiences of when they had been offered testing, how much information they had received before and after the test and how they had been informed. Being diagnosed with BC was a shock that created a need for information as well as an obstacle to receiving information. Trust in the healthcare personnel they met facilitated communication in this situation. Irrespective of how and when they were offered genetic testing, they all emphasised that it had been important to them. It was seen as important in their cancer treatment and an opportunity to protect their relatives from getting BC. Their combined experience illustrates the importance of simple and standardised routines to ensure that all eligible BC patients are offered genetic testing.

Identification of a *BRCA* mutation in a woman diagnosed with BC may have an impact on both surgical and oncological treatment decisions [2–8, 22]. There are therefore several medical arguments for offering BC patients genetic testing shortly after diagnosis, and genetic testing is increasingly being offered to patients at this point. Earlier studies suggested that, at the time of diagnosis or during treatment, BC patients are too emotionally overwhelmed to be able to make an informed decision about genetic testing [9, 10]. However, more recent studies have demonstrated that BC patients see the test as important for medical decision-making [13–15] and that offering testing shortly after diagnosis does not cause adverse psychological effects [11, 12]. In contrast to previous studies, the women interviewed for this study were not offered genetic counselling prior to testing. In addition, genetic testing was offered as part of regular clinical care and not as part of a research protocol. The participants described in detail their shock and fear upon being diagnosed with BC, how difficult it was to think clearly in this situation and how this made it difficult to take in and remember the information that was provided to them. However, where previous studies concluded that it would be too much to offer them testing at this point [9, 10], the women interviewed all emphasised that getting access to genetic testing had been important to them. It was an important and necessary part of their diagnosis and treatment, and it was perceived as an important way of helping their relatives.

Information was a recurrent theme among the women interviewed for this study. Some actively sought detailed information about their diagnosis and treatment, while others did not express the same need. Miller et al. have described how individuals have different approaches to seeking and processing information in challenging situations [23]. In their study, they differentiate between high and low monitors, where high monitors tend to seek more comprehensive and detailed information in cancer-related and other medical contexts than do low monitors [24]. High monitors tend to heighten threats in health information and worry about these threats for a longer period of time than low monitors, who tend to limit and distance themselves from the threatening information [24]. Previous research suggests that high monitoring seems to be related to a desire for more detailed information [25–27], participation in medical decision-making, and more question-asking and dominance [25]. High monitors also tend to be less satisfied with the information provided to them [28]. It is said that in a crisis it is important to facilitate a person’s understanding of the actual situation [29]. Women in this threatening situation are striving to gain control of the situation. According to Griffin [30], it is essential for individuals to feel that they have control over their own fate. This is also in line with Glanz et al. [31], who indicate that coping strategies may have a significant impact on psychological and physical health outcomes. For many of the participants in this study, the genetic test itself helped them gain control over the situation. It provided important information regarding their diagnosis and their relatives’ risk of cancer, and it helped them navigate this unfamiliar terrain. Surprisingly, despite being relieved by a normal test result, some of the women indicated that they sometimes wished that they had tested positive for a *BRCA* mutation. If they had tested positive, then they would have understood why they had been diagnosed with BC and they would also have been able to have a risk-reducing mastectomy. This inclination may reflect the underlying fear of recurrence which many BC survivors experience [32, 33].

The level of information the participants had received about genetic testing varied. Except for the two participants who had a general feeling of not receiving enough information during their treatment, most of the participants expressed satisfaction with the limited information they had received before the test, even though they had a hard time remembering the details. The participants regarded written information as helpful and necessary, but the results may indicate that most BC patients do not need detailed information about testing. The participants’ experiences were also largely affected by their trust in their healthcare providers and the way they communicated with them. This is in line with previous studies demonstrating that factual information regarding an illness may be less significant to mental well-being than the way the information is provided. According to Austin, affective outcomes, such as satisfaction from communicating, is regarded as more valuable than the actual information provided [34, 35]. It may therefore be important to the patient experience that clinicians be confident when providing information about genetic testing and that they adjust the level of information they provide according to the patient’s needs.

In a recent study from the UK, geneticists and non-genetics specialists were interviewed regarding their views on potentially mainstreaming genetic testing into the care of newly diagnosed BC patients [36]. It was pointed out that perceived workload associated with this practice, the relevance of the test result for their patients and the view of their profession’s responsibilities affected their view of mainstreaming genetic testing and could acts as barriers to their implementation. The participants in this study were interviewed about a future change of practice. We do not know to what extent these perceived barriers to mainstreaming genetic testing are relevant to the clinicians currently involved with diagnosis and treatment of BC in South-Eastern Norway; however, the participants’ variable experiences regarding how and when testing was offered may reflect variation in the routines for this healthcare service and genetic testing not being fully mainstreamed into clinical care at the time they were diagnosed and treated for BC. This is also illustrated and supported by the fact that as many as 27% of the participants reported that they had not been offered the test but had asked for it themselves. At the same time, several of the participants emphasised how important it is that BC patients be offered genetic testing and that a system be in place to ensure that they all get it. It has been pointed out that effective communication between specialties together with clear guidelines may contribute to overcoming potential barriers to implementing genetic testing in the regular clinical care of BC patients [36]. In the region of South-Eastern Norway, BC treatment is offered at eight hospitals. The medical genetics department with regional responsibility is located at OUH, the only hospital in the region that employs geneticists and genetic counsellors. Currently, there is limited regular contact between geneticists from OUH and surgeons and oncologists involved in BC diagnostics and treatment at the other hospitals in the region. Our combined findings may indicate that there is a need for continuous collaboration and communication between the medical genetics department at OUH and the other hospitals in order to identify possible challenges in implementing genetic testing in the medical treatment of BC patients and establish simple and robust routines for this healthcare service. Regular contact may also contribute to improving the level of knowledge about hereditary BC and genetic testing among the surgeons and oncologists in the region. Moreover, at the moment, the Norwegian criteria do not state at what point genetic testing should be mentioned to the patient during the diagnosis and treatment process. The patients’ varied experience in the timing of genetic testing may indicate a need for clarification in this regard.

### Methodological considerations

The interviews were conducted by two genetic counsellors with long-standing experience of clinical work with hereditary cancer. This may have enhanced their communication with the participants. The computer program NVIVO was used to organise and code the processing of data. This may strengthen the reliability of this study. The number of participants was limited. However, the themes identified may be relevant on a more general level than one woman’s experience [37]. All the women who were interviewed for this study wanted to be tested, and all but one had normal test results. We cannot exclude this having affected their experience of mainstreamed genetic testing. Our previous study on the rates of genetic testing in these two hospitals demonstrated that 96% of patients who were offered testing wanted to be tested [16]. As such an overwhelming majority of BC patients who are offered testing want to be tested, it is relevant to gather information about their experience to ensure that the healthcare service is offered in a way and at a time that meets their needs. However, interviewing patients who had been offered testing but declined may provide a more nuanced picture and should be addressed in future studies. Future studies should also address the experience of those who tested positive for a *BRCA1/2* mutation. All interviews were conducted two to three years after the participants were tested. Hence, we cannot exclude the potential of recall bias, and future studies may seek to interview patients who have been tested more recently.

## Conclusions

The aim of this study was to improve our knowledge about how BC patients who have been offered genetic testing by their treating surgeon or oncologist experience this healthcare service. The women interviewed had varied experiences of when and how they were offered testing, and 27% of them reported that they had asked for the test themselves. Irrespective of how testing was offered, and despite the shock and chaos that accompanied their BC diagnosis, they all emphasised that genetic testing was an important part of their diagnosis and treatment. Our mixed results indicate a need for continual collaboration between geneticists, surgeons, oncologists and laboratory specialists in order to establish simple and robust routines and ensure that all eligible BC patients are offered testing at a point when the test result can influence medical treatment. Regular contact may be helpful in identifying the challenges that clinicians face when implementing genetic testing in clinical care, while also potentially increasing knowledge and competence regarding hereditary BC and genetic testing among surgeons and oncologists.

## Data Availability

The datasets generated and analysed in the current study are available from the corresponding author upon reasonable request.
